# Prediction of Pancreatic Neuroendocrine Tumor Grading Risk Based on Quantitative Radiomic Analysis of MR

**DOI:** 10.3389/fonc.2021.758062

**Published:** 2021-11-17

**Authors:** Wei Li, Chao Xu, Zhaoxiang Ye

**Affiliations:** ^1^ Department of Radiology, Tianjin Medical University Cancer Institute and Hospital, National Clinical Research Center for Cancer, Key Laboratory of Cancer Prevention and Therapy, Tianjin’s Clinical Research Center for Cancer, Tianjin, China; ^2^ Department of Pancreatic Cancer, Tianjin Medical University Cancer Institute and Hospital, National Clinical Research Center for Cancer, Key Laboratory of Cancer Prevention and Therapy, Tianjin’s Clinical Research Center for Cancer, Tianjin, China

**Keywords:** grade risk, radiomic features, MR, prediction, PNETs

## Abstract

**Background:**

Pancreatic neuroendocrine tumors (PNETs) grade is very important for treatment strategy of PNETs. The present study aimed to find the quantitative radiomic features for predicting grades of PNETs in MR images.

**Materials and Methods:**

Totally 48 patients but 51 lesions with a pathological tumor grade were subdivided into low grade (G1) group and intermediate grade (G2) group. The ROI was manually segmented slice by slice in 3D-T1 weighted sequence with and without enhancement. Statistical differences of radiomic features between G1 and G2 groups were analyzed using the independent sample *t*-test. Logistic regression analysis was conducted to find better predictors in distinguishing G1 and G2 groups. Finally, receiver operating characteristic (ROC) was constructed to assess diagnostic performance of each model.

**Results:**

No significant difference between G1 and G2 groups (*P* > 0.05) in non-enhanced 3D-T1 images was found. Significant differences in the arterial phase analysis between the G1 and the G2 groups appeared as follows: the maximum intensity feature (*P* = 0.021); the range feature (*P* = 0.039). Multiple logistic regression analysis based on univariable model showed the maximum intensity feature (*P*=0.023, OR = 0.621, 95% CI: 0.433–0.858) was an independent predictor of G1 compared with G2 group, and the area under the curve (AUC) was 0.695.

**Conclusions:**

The maximum intensity feature of radiomic features in MR images can help to predict PNETs grade risk.

## Introduction

Pancreatic neuroendocrine tumors (PNETs), as the second most common epithelial neoplasm of the pancreas (1, 2), have increased significantly over the last decade (3). Based on histological differentiation (including mitoses and Ki-67 proliferation index), the WHO 2017 classification (4) has separated well-differentiated PNETs into three groups: low grade (G1), intermediate grade (G2), and high grade (G3). PNETs often cause severe morbidity due to excessive secretion of hormones (such as serotonin) and/or overall tumor mass, but in the clinic, lack of specific biomarkers inhibits early diagnosis (5). It was reported (6) that the PNETs grading was useful for therapeutic decisions and had a great impact on survival for PNETs (7). According to the different grades risk of PNETs, surgical resection or medical therapies should be performed for different patients (2). As the biological behavior of PNETs is relatively variable, pretreatment predictive aggressiveness of individual tumors is therefore very important in determining an efficient treatment strategy for patients to minimize harm from possible over- or undertreatment, especially for those with more advanced disease that cannot be resected (8).

MR imaging methods may help define the more appropriate treatment strategy for PNETs in a non-invasive way (2). In fact, previous studies have identified several traditional MR imaging features that could be potentially valuable for discrimination of tumor grades in PNETs (9–14), such as tumor sizes (15), irregular margins, and enhancement pattern (9, 16, 17); moreover, some authors reported that diffusion weighted imaging (DWI) in MR imaging might have the capability of roughly distinguishing high-grade PNETs from G1 tumors (16, 18–25).

However, now, except for these traditional MR features, especially in DWI that suggested the discrimination of tumor grades in PNETs, there are still no generally accepted quantitative guidelines to predict the PNETs grading. Radiomic analysis, as a more systematic approach, may provide more quantitative information regarding the discrimination of different biological behavior of PNETs (26), as it is able to identify voxel-level changes within PNETs. Several studies had focused on predictors of PNETs grades based on radiomic analysis just only in CT imaging (27, 28). There were few studies that focused on MR imaging based on radiomic analysis. Thus, our study presents the hypothesis that there may be some radiomic features in MR that can help to predict grades of PNETs. The present study aims to find the quantitative radiomic features for predicting grades of PNETs in MR images with pathological diagnoses using radiomic analysis.

## Materials and Methods

### MRI Examinations

All the MR examinations were performed using a 1.5T GE MRI scanner (SignaExcite HD, GE Healthcare, Milwaukee, WI, USA) equipped with eight channel phased-array coils, and the scanning parameters were as follows: T2-weighted MR images with respiratory-triggered fat-saturated fast spin-echo sequences for identifying the lesion’s location [TR/TE =7,500/86 ms; slice thickness = 7 mm; space gap = 1 mm; field of view (FOV) = 40×34 cm; matrix = 128 × 128 or 320×160]. An axial breath-hold T1-weighted 3D fat suppressed spoiled gradient-echo (GRE) sequence (liver acquisition with volume acceleration, LAVA) before contrast agent injection was used for dynamic contrast-enhanced imaging (TR/TE = 6.2/3.1 ms; flipangle = 12; FOV = 315×360; matrix = 256×256; section thickness = 4 mm). Contrast images were acquired during the arterial (20 s delay), portal venous (60 s delay), and equilibrium phases (180 s delay), and the contrast agent was applied with a bolus injection of 0.1 mmol/kg body weight of gadopentetate dimeglumine (Magnevist, Bayer Schering, Berlin, Germany).

### Delineation of ROI

The ROI in the present study was manually delineated and segmented on the MR images. The lesions were manually delineated and segmented slice by slice on the non-enhanced and the enhancement T1 images for ROIs of the radiomic analysis.

Finally, the seed ROIs were checked in each lesion of each patient by another radiologist to ensure that the ROI in each patient satisfied the lesion boundary definition.

### Computerized Radiomic Analysis Based on the ROI

The radiomic analysis was performed using the 3D slicer software (Version 4.6.2; Surgical Planning Laboratory, Brigham and Women’s Hospital, Harvard Medical School, Boston, MA, USA) (http://www.slicer.org) (29). Then, the radiomic features were calculated and extracted automatically using the module called “Heterogeneity CAD”. The radiomic features (a total of 44 features, shown in **Table S1** of the **Supplementary Materials**) were divided into three categories, including the following (1): first-order and distribution statistics (2), shape and morphology metrics (3), the gray-level co-occurrence matrix (GLCM).

The overall procedure of this analytical scheme was performed by two radiologists (with 9 and 6 years’ experience in abdominal MR imaging, respectively). Finally, we computed the means of each of the MR radiomic feature values measured by the two independent observers. The interobserver agreement regarding the radiomic features of the ROIs was calculated using the interclass correlation coefficient analysis (ICC) using the SPSS software.

### Radiomic Statistical Analysis

All statistical analyses were conducted with the Statistical Package for the Social Sciences version 19.0 (IBM Corp. IBM SPSS Statistics for Windows). Interobserver agreement was assessed using the interclass correlation coefficient analysis (ICC). ICC value of ≤0.4 indicated poor agreement; 0.41–0.6, moderate agreement; 0.61–0.80, substantial agreement; 0.81–1.00, excellent agreement. Continuous variables were expressed as mean ± SD, and statistical differences between G1 group and G2 group were analyzed using the independent sample *t*-test for differences in the radiomic features. The data was corrected by Bonferroni’s approach (P < 0.05) with two-sided to control for the type 1 errors.

Logistic regression analysis was conducted to find better predictors in distinguishing G1 group and G2 group. Features with *p* value of <0.05 in univariable model were entered into the multiple logistic regression analysis. The stepwise model selection using forward.LR (likelihood ratio test) methods was used to select the final predictive model. Receiver operating characteristic (ROC) curves for each model were constructed. The area under the curve (AUC) and its 95% confidence interval estimated using DeLong’s method were calculated to evaluate the performances of the regressive models. A *p* value of <0.05 was considered a significant difference.

## Results

### Patients Population

The study was approved by the Medical Research Ethics Committee and the Institutional Review Board. The patients in our study underwent preoperative upper abdominal MRI at our institution between January 2011 and January 2018. The inclusion criteria for the PNETs patients in our study were as follows: (1) patients with a surgery and pathological diagnosis of pancreas tumor, and graded by the European Neuroendocrine Tumor Society (ENETS), WHO 2017, based on mitotic count and Ki-67 index; (2) diagnostic MRI scans before surgery; (3) MRI images with a slice thickness of 5 mm or less.

Ultimately, there were 48 patients with 51 lesions (mean age, 50.4 years; age range, 16–74 years) who enrolled in our study. Based on systems of grading for PNETs, in our study, the lesions were subdivided into low grade (G1) group and intermediate grade (G2) group. Twenty-six of the patients (51.0%) had G1 lesions, and 25 (49.0%) had G2 lesions. The patients’ basic clinical characteristics and the MRI features of the PNETs are shown in **Table 1**.

**Table 1:** The basic clinical characteristics of patients and the MRI features with WHO tumor grade of pancreatic neuroendocrine tumors. .

Features	Tumor grade	
	G1 (n = 26)	G2 (n = 25)
**Female**	18 (69.2%)	15 (60.0%)
**Male**	8 (30.8%)	10 (40.0%)
***Age (years)***		
Mean	53.34	49.1
Range	16–67	26–74
Standard deviation	11.8	11.7
**Tumor location**		
Pancreas head	8 (30.8%)	6 (24.0%)
Pancreas body	12 (46.2%)	12 (48.0%)
Pancreas tail	6 (23.0%)	7 (28.0%)
**Tumor sizes (cm)**		
Mean ± SD	3.4 ± 2.1	3.6 ± 2.5
Range	1.0–10.3	1.0–10.5
Lesions (<2 cm)	9	6
**Tumor pattern**		
Pancreatic duct dilatation	7	6
Chronic atrophic pancreatitis	3	5
Vascular involvement	6	12
Fibrosis on the surrounding pancreatic parenchyma	0	2
Ki-67 index (%)	<2	3–10
**Lymphadenopathy**	0	4
**Synchronous liver metastases**	3	3

The interobserver agreement regarding the radiomic features of the PNETs ROI was generally acceptable (the value ranged from 0.717 to 0.986).

### Significant Radiomic Features Differences of Tumor Grades

In the comparison of the radiomic analyses in non-enhanced T1 images, there was no significant difference between G1 group and G2 group (*P* > 0.05). However, significant differences only in the arterial phase analysis of enhanced images between the G1 group and the G2 group appeared in the radiomic features as follows: maximum intensity (*P* = 0.021, ICC = 0.807); range (*P* = 0.039, ICC = 0.908) (**Table 2**).

**Table 2:** Significant differences in the radiomic features between PNETs G1 group and PNETs G2 group.

Texture features	*G1 group (n=26)*	*G2 group (n=25)*	P value*
Maximum intensity	1,868.73 ± 489.34	1,595.80 ± 298.50	0.021
Range	1,284.88 ± 577.693	997.68 ± 358.45	0.039

Data are mean ± standard deviation

*Independent sample t test.

PNETs, pancreatic neuroendocrine tumors.

### Logistic Regression Analysis and ROC Analysis for the Prediction of Tumor Grades

**Figure 1:**
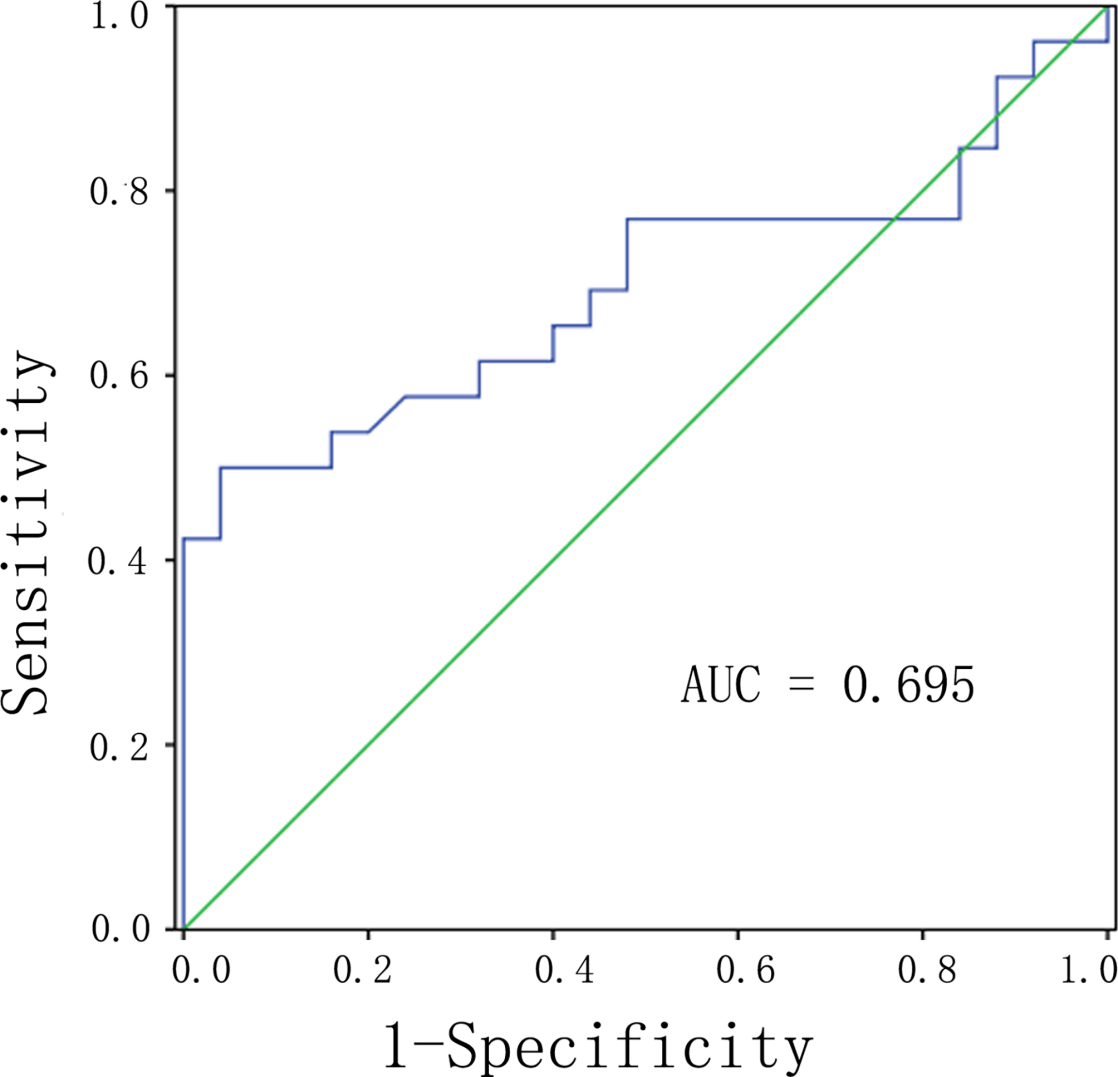
ROC analysis of the significant differences of radiomic analysis between G1 and G2 of PNETs. Abbreviations: PNETs, pancreatic neuroendocrine tumors.

We used the maximum intensity feature and the range feature above as input variables for multiple logistic regression analysis. Logistic regression analysis revealed that the maximum intensity feature (*P* = 0.023, OR = 0.621, 95% CI: 0.433–0.858) was an independent predictor of G1 group compared with G2 groups (**Table 3**). The area under the curve (AUC) was 0.695 (95% CI: 0.543–0.846; P = 0.017) with a sensitivity and specificity of 50.0 and 92.0%, respectively (**Figure 1**).

**Table 3:** Logistic regression analysis of the radiomic features between grade G1 and G2 group of PNETs.

Feature	Regression coefficients	P value	OR	95% CI
Maximum intensity	−0.103	0.023	0.621	0.433~0.858

PNETs, pancreatic neuroendocrine tumors.

## Discussion

PNETs grades were significant for tumor treatment. The present study used MR imaging to predict the grades based on radiomic analysis, and it showed that the maximum intensity feature in the arterial phase T1 weight images of MR could be an independent predictor of G1lesions compared with G2 lesions of PNETs.

Radiomic analysis has been suggested a useful tool for the quantitative assessment of tumor heterogeneity (30, 31). The heterogeneity within tumors is associated with histopathologic grade and prognosis of tumors, which can reflect the intrinsic biologic aggressiveness of tumors (32–34). Several previous (35) studies have shown that the assessment of tumor heterogeneity had an important value for diagnosis, grading, prognosis, and treatment monitoring. MR imaging of tumor may provide a non-invasive assessment of tumor heterogeneity and may represent a valuable non-invasive tool in predicting the grading of PNETs and help for aggressiveness and prognosis of PNETs.

There have been several studies that investigated the CT radiomic characteristics, which can predict the grades of PNETs (27, 28). It was shown that the sphericity feature of radiomic variables on arterial 2D analysis of CT could be significant predictors between grade 2/3 and grade 1 (28). And also, the entropy feature of radiomic features in CT images was found as an independent predictor of PNETs grade (27).

However, there were few studies focused on radiomic analysis in MR images, although MR scanning can provide more sensitivity for structural investigation and higher soft tissue contrast resolution that makes it superior to CT in detecting PNETs, especially small tumors. Several studies (36, 37) about MR data applied to PNETs focused on ADC map, and it was found that the entropy and the kutosis features of histogram analysis, which was a part of radiomic analysis in ADC images of MR, could predict the G1 compared with the G2 of PNETs (36). Besides the most studies about MR features in ADC map associated with PNETs grades, radiomic analysis of PNETs in T1 weight images of MR were scarce, which could provide some more different information of MR images than ADC map. In a recent study it suggested that MRI radiomic score showed a significant association with the grades of PNETs (38), and another study showed the developed radiomics model using non-contrast MRI could help differentiate G1 and G2/3 tumors (39); both of that suggested radiomic MRI may be used as a valuable non-invasive tool for differential PNET grading.

In our study, we found that the maximum intensity feature of radiomic features was an independent predictor of G1 lesions compared with G2 lesions in the arterial phase T1 weight images of MR, although statistical significance was not found in the non-enhanced T1 images of MR. The results in our study showed G1 PNETs had significant differences on the maximum intensity feature of radiomic features after enhancement compared with higher grade tumors. It also coordinated with the previous report, which demonstrated that G1 PNETs were enhanced more prominently than higher-grade tumors in MR imaging (1). The lower-grade PNETs showed significantly increased tumor blood flow than higher-grade lesions (40). In the present study, the maximum intensity feature means the value of the voxels in the image ROI with the greatest value, which is thought to reflect tissue heterogeneity quantitatively by images. The maximum intensity feature of radiomic features may reflect the differences of blood flow within the tumor by different values in the image, and also reflect the tumor heterogeneity.

The results of our study may contribute to the development of predicting models that combine quantitative and qualitative radiomic features of imaging and traditional MR image feature predictors.

In the last couple of decades, the introduction and development of the endoscopic ultrasonography (EUS) opened a new era of diagnosis and treatment of PNETs, which had become a very useful imaging modality to evaluate pancreatic lesions. Contrast-enhanced EUS is helpful in categorizing small hypervascular PNETs (41), and studies showed that EUS was superior for the detection of PNETs lesions smaller than 2 cm (42–47). Its sensitivity was equal to MRI for the detection of PNETs. Other befits of EUS include the detection of lymph node involvement and vascular invasion. In the last few years, as the development of properly designed needles for EUS-guided fine-needle biopsy (EUS-FNB), the EUS-FNB was more important in the evaluation of suspected PNETs, especially in small (with a diameter smaller than 2 cm), non-functioning PNETs (45–47), which showed stronger and more accurate correlation for Ki-67 values with surgical specimens. MRI may not provide more accurate cytological information inside the tumor than EUS-FNB; however, it is a non-invasive technology compared with EUS-FNB, which can quantitatively assess tumor heterogeneity. Also, each imaging method is not perfect and needs to be combined in future applications.

Our study had several limitations. First, as it was of retrospective design and PNETs are rare tumors. The patients in our study included only 48 patients with 51 lesions with G1or G2 PNETs. Second, in our study, tumor segmentation was manually performed and which may be influenced by some manual errors, as well as affecting the radiomic analysis results. So robust automatic boundary extraction method should be further developed for accurate ROI lesions. Nevertheless, additional long-term studies are needed to validate the results in larger population and in other sequences of MR images.

## Conclusions

In conclusion, radiomic analysis of MR is helpful for the prediction of PNETs grade. The maximum intensity feature can help to identify the G1 PNETs from G2 PNETs on the arterial phase images of MR, which may be also applied to early recurrence or progression after surgical resection of PNETs in the further study.

## Data Availability Statement

The original contributions presented in the study are included in the article/**Supplementary Material**. Further inquiries can be directed to the corresponding author.

## Ethics Statement

The studies involving human participants were reviewed and approved by the Medical Research Ethics Committee and the Institutional Review Board of Tianjin Medical University Cancer Institute and Hospital. Written informed consent for participation was not required for this study in accordance with the national legislation and the institutional requirements.

## Author Contributions

WL made contributions to study concepts and design, literature research, statistical analysis, manuscript preparation, and manuscript editing. CX made contributions to clinical studies. ZY contributed as guarantor of the integrity of the entire study. All authors contributed to the article and approved the submitted version.

## Conflict of Interest

The authors declare that the research was conducted in the absence of any commercial or financial relationships that could be construed as a potential conflict of interest.

## Publisher’s Note

All claims expressed in this article are solely those of the authors and do not necessarily represent those of their affiliated organizations, or those of the publisher, the editors and the reviewers. Any product that may be evaluated in this article, or claim that may be made by its manufacturer, is not guaranteed or endorsed by the publisher.
