# Supplementary material for: Prediction of Pancreatic Neuroendocrine Tumor Grading Risk Based on Quantitative Radiomic Analysis of MR
Source: Front Oncol. 2021 Nov 17;11:758062. doi: 10.3389/fonc.2021.758062 (PMC8637752; doi:10.3389/fonc.2021.758062)
Supplement: Supplementary file 1 [file DataSheet_1.pdf]

Supplementary Materials

Tables

Table S1 Quantitativeradiomic feature categories and feature names.

Feature category	Feature numbers and Feature names					
First-order and distribution statistics	F1	Gray Levels	F7	Mean Intensity	F13	Skewness
	F2	Voxel Count	F8	Median Intensity	F14	Kurtosis
	F3	Energy	F9	Range*	F15	Variance
	F4	Entropy	F10	Mean Deviation	F16	Uniformity
	F5	Minimum Intensity	F11	Root Mean Square		
	F6	Maximum Intensity*	F12	Standard Deviation		
Shape and morphology metrics	F17	Volume(mm^3)	F20	Compactness 1	F23	Sphericity
	F18	Surface Area(mm^2)	F21	Compactness 2	F24	Volume cc
	F19	Surface: Volume Ratio	F22	Spherical Disproportion	F25	Maximum3D Diameter
Gray-level co-occurrence matrix	F26	Autocorrelation	F33	Homogeneity 2	F40	IDN
	F27	Cluster Prominence	F34	IDMN	F41	Contrast
	F28	Cluster Shade	F35	Energy (GLCM)	F42	Dissimilarity
	F29	Cluster Tendency	F36	Inverse Variance	F43	Sum Average
	F30	Variance(GLCM)	F37	Maximum Probability	F44	Entropy(GLCM)
	F31	Difference Entropy	F38	Sum Entropy		
	F32	Homogeneity 1	F39	Sum Variance		

Abbreviations: GLCM, gray-level run length matrix; IDMN, inverse difference moment normalized; IDN, inverse difference normalized; Volume cc , volume in cubic centimeters;

Note: *, statistically significant to distinguish PNETs G1 group and G2 group in the present study.

Table S2 The radiomic features between PNETs G1 group and PNETs grade G2 group without significant differences.

Feature category	Texture Feature numbers and names	*G1 group(n=26)*	*G2 group(n=25)*	*Pvalue**
First-order and distribution statistics	F1 Gray Levels	580.080±362.277	492.760±361.294	0.393
	F2 Voxel Count	1921.960±1960.772	2356.400±3108.648	0.052
	F3 Energy	4195231026.270 ±4665648302.149	3980102996.520±5908516952.534	0.886
	F4 Entropy	4303.0610± 5964.461	7195.740±12040.743	0.279
	F5 Minimum Intensity	583.850±302.673	598.120±328.118	0.872
	F7 Mean Intensity	1382.424±327.508	1230.731±271.429	0.078
	F8 Median Intensity	1403.520±331.174	1253.880±278.966	0.088
	F10 Mean Deviation	160.275±82.228	122.592±52.097	0.057
	F11 Root Mean Square	1402.197±324.242	1243.556±270.564	0.064
	F12 Standard Deviation	209.097±98.311	162.897±70.326	0.060
	F13 Skewness	-0.832±1.031	-0.817±0.817	0.955
	F14 Kurtosis	2.299±3.910	1.910±2.791	0.685
	F15 Variance	53015.256±49965.258	31283.531±29262.953	0.065
	F16 Uniformity	12135.120±19200.901	29679.440±61066.040	0.169
Shape and	F17 Volume (mm^3)	2194.515±2045.288	2601.614±3330.003	0.600

morphology metrics	F18	Surface Area(mm^2)	2267.663±2030.823	2618.356±3165.486	0.638
	F19	Surface: Volume Ratio	1.098±0.139	1.107±0.144	0.812
	F20	Compactness 1	6.424±2.415	6.456±3.084	0.967
	F21	Compactness 2	0.085±0.069	0.096±0.085	0.620
	F22	SphericalDisproportion	0.001±0.001	0.001±0.001	0.588
	F23	Sphericity	0.411±0.112	0.421±0.129	0.760
	F24	Volume cc	2.194±2.045	2.601±3.330	0.600
	F25	Maximum 3DDiameter	39.727±22.446	40.284±26.395	0.936
Gray-level co-occurrence matrix	F26	Autocorrelation	141904776.304±231096591.959	348762227.553±1032898496.483	0.324
	F27	Cluster Prominence	1261036132837760±2809491732923770	6237395056682930±21279759420388600	0.243
	F28	Cluster Shade	784071718991.580±1497618172542.830	2824086323165.350±9234855340226.810	0.272
	F29	Cluster Tendency	562702872.303±916263233.615	1375668260.843±4078934683.157	0.327
	F30	Variance(GLCM)	143563464.657±233594224.126	350094746.607±1035423504.874	0.326
	F31	Difference Entropy	2640.606±3334.365	3849.353±6191.683	0.387
	F32	Homogeneity 1	39.274±37.173	54.006±66.214	0.330
	F33	Homogeneity 2	15.571±14.562	22.135±26.883	0.281
	F34	IDMN	573.704±593.610	706.489±944.711	0.549
	F35	Energy (GLCM)	590.017±617.927	764.636±1059.850	0.474

F36	Inverse Variance	17.122±15.431	23.875±28.359	0.293
F37	Maximum Probability	0.630±0.185	0.723±0.299	0.188
F38	Sum Entropy	-918.551±1299.646	-1628.408±2813.395	0.250
F39	Sum Variance	6751764554.030±16048471582.693	186300600419.810±694630463112.622	0.194
F40	IDN	573.704±593.610	706.489±944.711	0.549
F41	Contrast	3321913.917±5257050.818	2675141.347±5779167.800	0.678
F42	Dissimilarity	28742.150±35467.334	29055.710±48191.149	0.979
F43	Sum Average	493545.479±671015.654	808270.233±1870436.378	0.424
F44	Entropy (GLCM)	-2566.859±3588.530	-4314.249±7271.212	0.279
